# Imaging Viral Infection by Fluorescence Microscopy: Focus on HIV-1 Early Stage

**DOI:** 10.3390/v13020213

**Published:** 2021-01-30

**Authors:** Soumajit Mukherjee, Emmanuel Boutant, Eleonore Réal, Yves Mély, Halina Anton

**Affiliations:** 1 Department of Chemistry and Biochemistry, Mendel University in Brno, Zemedelska 1, CZ-613 00 Brno, Czech Republic; soumajit.mukherjee@mendelu.cz; 2 Laboratory of Bioimaging and Pathologies, CNRS UMR 7021, Faculty of Pharmacy, Strasbourg University, 74, route du Rhin, F-67401 Illkirch, France; emmanuel.boutant@unistra.fr (E.B.); eleonore.real@unistra.fr (E.R.); yves.mely@unistra.fr (Y.M.)

**Keywords:** HIV-1, fluorescence microscopy, virus labeling

## Abstract

During the last two decades, progresses in bioimaging and the development of various strategies to fluorescently label the viral components opened a wide range of possibilities to visualize the early phase of Human Immunodeficiency Virus 1 (HIV-1) life cycle directly in infected cells. After fusion of the viral envelope with the cell membrane, the viral core is released into the cytoplasm and the viral RNA (vRNA) is retro-transcribed into DNA by the reverse transcriptase. During this process, the RNA-based viral complex transforms into a pre-integration complex (PIC), composed of the viral genomic DNA (vDNA) coated with viral and host cellular proteins. The protective capsid shell disassembles during a process called uncoating. The viral genome is transported into the cell nucleus and integrates into the host cell chromatin. Unlike biochemical approaches that provide global data about the whole population of viral particles, imaging techniques enable following individual viruses on a single particle level. In this context, quantitative microscopy has brought original data shedding light on the dynamics of the viral entry into the host cell, the cytoplasmic transport, the nuclear import, and the selection of the integration site. In parallel, multi-color imaging studies have elucidated the mechanism of action of host cell factors implicated in HIV-1 viral cycle progression. In this review, we describe the labeling strategies used for HIV-1 fluorescence imaging and report on the main advancements that imaging studies have brought in the understanding of the infection mechanisms from the viral entry into the host cell until the provirus integration step.

## 1. Introduction

The Human Immunodeficiency Virus 1 (HIV-1) life cycle begins with the viral entry that is triggered by the binding of the viral envelope glycoproteins to the CD4 receptors and CCR5 or CXCR4 co-receptors of the host cell. This initial binding leads to the fusion of the viral and cellular membranes and subsequently to the release of the viral core in the host cell cytoplasm [1,2]. Once in the cytoplasm, the virus hijacks the cell transport machinery and reaches the perinuclear area via the microtubule network [3,4,5]. The RNA-based reverse transcription complex (RTC) is transformed into the integration competent preintegration complex (PIC), composed of double-stranded (dsDNA) viral genome and several viral and cellular proteins. During this time, the viral capsid is partly disassembled, and the PIC is imported into the nucleus and integrates the viral DNA into the host cell genome [6,7,8,9].

During the last two decades, fluorescence microscopy has become a powerful tool for deciphering the key steps of the HIV-1 life cycle. In addition to the biochemical and molecular biology approaches, imaging of the virus at the level of individual viral particles has helped to identify the key steps and interactions leading to productive infection. Time-lapse microscopy in living cells has provided critical information on the kinetics of infection, the intracellular transport mechanism [3,4,5,10,11] and has unveiled unexpected steps such that the prolonged viral docking at the nuclear envelope which is crucial for its nuclear entry [12,13]. Smart labeling strategies have been developed to visualize membrane fusion [14], loss of viral core integrity [15,16], uncoating [13,17,18,19], release of viral proteins [20,21,22], and remodeling of the viral complex. Original tools have been developed to visualize the viral genome and to follow the nuclear trafficking of the PIC leading to integration.

The aim of this review is to describe selected labeling strategies and fluorescence microscopy-based studies that led to significant advancements in the understanding of the successive steps of the early phase of HIV-1 infection. This complex and highly debated subject is covered by abundant literature that is beyond the scope of this review. It is not our goal to answer or take a position in these controversies, but much more to highlight how fluorescence-microscopy-based approaches, complementary to molecular biology and biochemical techniques, help to solve unanswered questions. Presented tools are of interest not only for studies on HIV-1 life cycle but also on other biological related questions and could thus inspire new research projects.

## 2. Fluorescent Labeling of HIV-1

**Figure 1:**
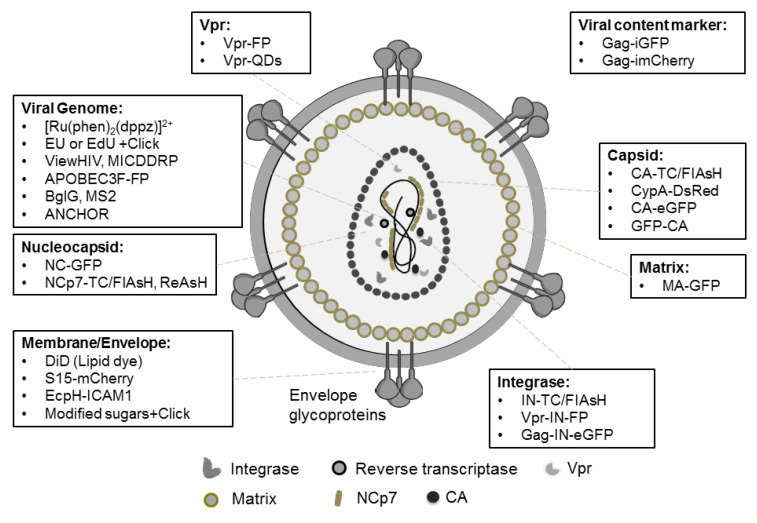
Reported strategies for fluorescent labeling of different Human Immunodeficiency Virus 1 (HIV-1) components (see Table 1 for details).

Historically, the first fluorescence microscopy observations of HIV-1 viral proteins have been made in infected cells from patients, immunolabeled using the monoclonal antibodies against Gag [23,24,25]. Virus labeling by fusion of HIV-1 viral proteins to fluorescent proteins (FPs) has been reported later. Incorporating FPs in the virion is tedious because of their large size (for example, the size of GFP is ~27 kDa) and potential oligomerization properties that might interfere with the viral assembly. Introduction of an FP can also perturb the function of the viral protein and alter the morphology and infectivity of the viral particles. Despite these limitations, various labeling strategies for different viral structures have been successfully developed (summarized in Table 1 and Figure 1).

The first fluorescently labeled HIV-1 viruses used for imaging contained green fluorescent protein (GFP) fused to the N-terminus of the Viral protein r (Vpr) [3,26]. The fusion of an FP with one of the Gag domains leading to the release of a chimeric protein upon maturation was more laborious. Direct fusion with an FP has only been reported for matrix (MA) [27] and more recently for capsid (CA) protein [18,28]. Integrase (IN)-GFP was incorporated into viral particles by its fusion to the C-terminus of the Gag protein (so called Gag-IN-GFP). In this construct, a protease cleavage site was inserted between Gag and IN to ensure the release of IN-GFP upon maturation [29]. A smart alternative strategy takes advantage of Gag-p6 mediated incorporation of Vpr during viral budding. The best-known example is the production of IN-GFP containing HIV-1 virus by incorporation of Vpr-IN-GFP in trans. A protease cleavage site inserted between Vpr and IN facilitates the IN-GFP release upon viral maturation [30,31].

Different teams have produced doubly labeled viral particles to study viral disassembly by monitoring the loss of signal of the released component relative to the signal of the marker remaining in the viral core. This approach has been successfully applied to studies of viral fusion by monitoring the loss of virus membrane labeling resulting from its transfer to lipids of the host cell plasma membrane. Similarly, the loss of capsid labeling was used to monitor the uncoating process. Production of doubly stained viral particles by independently incorporating the two markers leads to heterogeneous populations that contain different proportions of the two labels thus preventing ratiometric studies. To avoid this bias the doubly labeled mCherry-2CL-YFP-Vpr construct, containing two protease cleavage sites (CL) (SQNY/IRKVL), has been designed [32]. This construct is incorporated into the nascent virions via Vpr interaction with Gag. During the maturation process, the viral protease cleaves the two CL sites, resulting in viral particles that contain mCherry as a viral content marker and the viral core labeled by YFP-Vpr. Importantly, the two labels are always present at a 1:1 ratio.

Ingenious constructs called internal GFP (iGFP) or internal mCherry (imCherry) were designed as markers of the viral content [33,34]. In these constructs, the FPs are inserted between the CA and MA domains of Gag and flanked by protease cleavage sites, so that they are released upon maturation. A fraction of FP molecules stays inside the viral membrane as an intravirion fluid marker and is released upon membrane fusion, while the rest of the FPs are enclosed within the viral capsid shell and allows observing the capsid opening.

In parallel, labeling systems with smaller genetic tags and fluorescent markers have been implemented. For example, the tetracystein tag (TC-tag; molecular weight = 585 Da), that binds FlAsH or ReAsH fluorophores, has been successfully fused to CA, IN, and Nucleocapsid protein 7 (NCp7) without drastically compromising the viral infectivity [10,22,35,36].

Cellular proteins such as APOBEC3F (AF3 apolipoprotein B mRNA editing enzyme catalytic subunit 3F) or Cyclophilin A (CypA, a peptidyl-prolyl isomerase) that are incorporated into viral particles have been fused to FPs to label the viral core and viral capsid respectively [17,37].

The viral genome has been detected by fluorescence in situ hybridization (FISH) and by the more sensitive branch-chain (bDNA) FISH [38]. bDNA-FISH uses a set of small probes that hybridize with the target sequence and are detected by a branched amplification system. The latter consists of a pre-amplifier probe that binds several “amplifiers,” labeled by multiple fluorophores. ViewHIV technology combines bDNA with immunodetection of CA [38]. Multiplex immunofluorescent cell-based detection of DNA, RNA, and protein (MICDDRP) is a bDNA FISH technique combined with immunofluorescence. By using specific probes for vRNA, vDNA, nascent RNA transcripts, and also spliced and unspliced RNA, MICDDRP allows the visualizing of the viral genome and proteins all over the viral life cycle [39].

Alternatively, vRNA or vDNA can be labeled by incorporating modified nucleotides (ethynyl uridine or deoxy ethynyl uridine) during viral production or at the phase of reverse transcription (RT), respectively. These nucleotides are subsequently labeled by click reaction with fluorophores containing a picolyl azide group [16,40,41]. Click labeling is also used to stain modified sugars incorporated in the viral glycoproteins during the production step [42].

Finally, the vRNA has been visualized by using BglG and MS2 systems. These approaches, especially well suited for live-cell imaging, are based on high affinity binding of *Escherichia coli* BglG protein or bacteriophage coat protein MS2 to specific RNA sequences. BglG or MS2 are expressed in infected cells as fluorescent chimeras and bind to their target sequences inserted into the viral genome [12,43]. Similarly, the ANCHOR technology has been developed to visualize vDNA by inserting into the viral genome the ANCH3 sequence, which is specifically recognized by the fluorescent chimera of OR proteins, derived from bacterial parB protein. This technique enables imaging the vDNA in nuclei of live cells during several days. Moreover, it is compatible with sample preparation for transmission electron microscopy (TEM) [7].

Development of the above-mentioned labeling strategies has initiated a variety of microscopy-based studies that have enlightened important steps of the early phase of the HIV-1 viral cycle. In the next chapters we will describe the main advances brought by the imaging studies on viral entry into the host cell, cytoplasmic trafficking, remodeling of the viral core, capsid disassembly, nuclear entry and nuclear fate of the viral complexes.

## 3. Viral Entry

The entry of the virus into the host cell is the first step of the early phase of HIV-1 replication. It is initiated by the binding of the viral envelope glycoproteins (gp120 and gp41) to cellular CD4 receptor and CCR5 and CXCR4 co-receptors. These interactions trigger a sequence of conformational changes leading to the insertion of the fusion peptide located at the N-terminus of gp41 into the host cell membrane, which in turn creates a fusion pore permitting the release of the viral core into the host cell cytoplasm (for a detailed review, see [1]). After binding to the cell surface, the viral membrane can fuse with the host cell plasma membrane or/and the viral particles can be internalized via endocytosis and subsequently released into the host cell cytoplasm by endosomal fusion. It seems that the virus enters via both pathways, but it remains unclear which of these pathways leads to productive infection. Microscopy studies, employing β-lactamase (BlaM) assay of viral entry and/or tracking of individual viruses, brought critical, though contradictory, information to this debate [2,14,51]. BlaM technique relies on the incorporation of BlaM into the virus by Vpr. During the fusion, the BlaM-Vpr is released into the host cell cytoplasm, where it cleaves its fluorescent substrate CCF2, loaded into the cell. The fusion is detected by a spectral shift of CCF2 [52,53]. This approach provides information about the global fusion efficacy. In contrast, single virus tracking of fluorescent viruses enables to identify individual fusion events and to study their location and kinetics.

### 3.1. Fusion with the Plasma Membrane

An early pioneering study on the onset of HIV-1 infection by Stauber et al. [26] presented a confocal microscopy investigation of GFP-Vpr containing HIV-1 virions in HeLa and Jurkat cells. The labeled virions entered the cells within a few minutes and migrated into the cell cytoplasm but their fluorescence faded before reaching the cell nucleus. As in the cytoplasm, the GFP-Vpr spots did not co-localize with endosomal markers (clathrin or transferrin receptors), the authors concluded that the virions entered the cells after fusion with the plasma membrane [26].

Tracking studies of doubly labeled viruses offered an elegant approach to monitor the viral entry. By imaging quantum dots (QDs) containing viral particles with DiD-labeled viral membranes, the group of Zongqiang Cui observed that in CD4+ T lymphocytes and CEM-ss, the DiD and QD fluorescence signals separated at the cell surface. The DiD signal disappeared, while the QDs moved rapidly into the host cell cytosol, suggesting viral entry after fusion with the plasma membrane [54].

The fusion with the plasma membrane was also investigated in CD4+ cells by Herold et al. [55]. By imaging MA-FP or eGFP-Vpr containing HIV-1 viruses in the cell cytoplasm, these authors showed that the viral particles bound to CD4 receptors are internalized via clathrin dependent endocytosis. However, by measuring the efficacy of the viral entry by the BlaM assay, they showed that inhibition of endocytosis did not affect the viral release and infection. Moreover, by incubating the infected cells at temperatures blocking fusion but not endocytosis, in the presence of fusion inhibitors, they showed that regardless of the viral strain and the envelope protein (Env or VSV-G), the virus present in the endosomes did not contribute to the infection in T lymphoblastic (Sup T1-R5) cells, CEM-ss cells, and in primary human CD4+ T lymphocytes. The authors concluded that HIV-1 entry seems to occur mainly via fusion with the plasma membrane in these cells [55].

### 3.2. Endocytosis Mediated Entry

In one of the first HIV-1 entry studies, Campbell et al. [48], developed the S15-mCherry membrane marker as a tool to monitor the separation of the viral core from the viral membrane during fusion. Analysis of the loss of mCherry signal from Vpr-eGFP-containing VSV-G pseudotyped virions (which enter the cell via endocytosis) revealed that already by 1hour post-infection (h.p.i), 68% of cytoplasmic virions have lost the mCherry staining, indicating rapid release of the viral cores from the endosomes [48].

**Figure 2:**
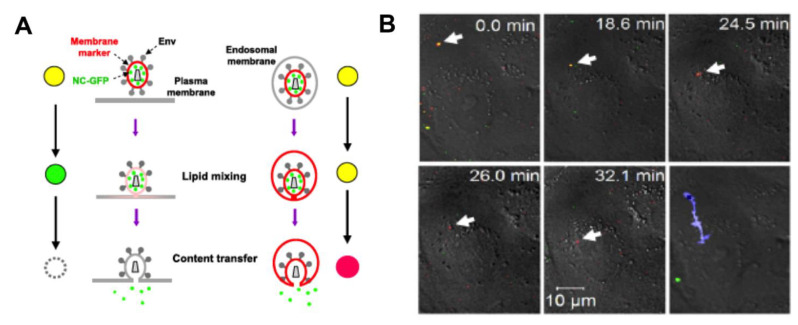
Fluorescence microscopy imaging of viral fusion: (**A**) Schematic of doubly labeled HIV-1 viral particles entering into the cell by fusion with the plasma membrane (left) or with the endosomal membrane (right). (**B**) A complete fusion of HIV-1 (JRFL strain) with endosomal membrane is evidenced by the loss of the green fluorescence signal due to the content release, and the incorporation of the red membrane stain into the endosomal membrane. The blue trace on the last image represents the intracellular trajectory of a viral particle. (Reproduced with permission from [45], copyright 2009, Elsevier).

The team of Melikyan developed several types of fluorescently labeled viral models to investigate HIV-1 cellular entry (reviewed in [2,14]). Miyauchi et al investigated the location of HIV-1 fusion in HeLa-derived cells. By monitoring the efficacy of the viral release by the BlaM assay while inhibiting fusion either at the plasma membrane, or in the whole cell, they identified endosomal fusion as a major cell entry pathway. To image the fusion events at the level of individual viral particles, they used MLV pseudoviruses with HIV-1 Env proteins. NC-GFP was used as a label of viral content and the viral membrane was stained by DiD (Figure 2A) [45]. By measuring the loss of red and green fluorescence signals the authors distinguished direct fusion with the plasma membrane (loss of the red signal followed by cytoplasmic diffusion of the green spot) from fusion with the endosomal membrane (resulting in the retention of the red lipid dye in the endosomal membrane upon release of the green viral content marker). Figure 2B shows the time-lapse images and the trajectory of a doubly labeled HIV-1 particle that has entered the cell cytoplasm and lost the green signal, demonstrating its fusion with endosomes. Distinct observations were made for viruses with JRFL or HXB2 envelopes. While the majority of JRFL pseudoviruses fused with the plasma membrane, HXB2 pseudoviruses released their content from endosomes ~30 min after their internalization. In T lymphoblastic CEMss cells, the viruses fused with endosomes, but some remained blocked at the stage of partial fusion at the plasma membrane. The lipid transfer at the cell surface occurred almost instantaneously after the onset of the infection, while the endosomal fusion was delayed by ~10 min. Finally, the HIV-1 fusion and infection were inhibited by a dynamin inhibitor dynasore, supporting the endosomal entry [45].

The HIV-1 trafficking in endosomal compartments has been further imaged using a Gag-mCherry containing HIV-1viruses with their surface labeled by the pH-sensitive GFP Ecliptic pHluorin (EcpH) [50]. The EcpH sensor was inserted into the viral membrane by its fusion to ICAM1 (intercellular adhesion molecule 1), which is incorporated into the HIV-1 virions during viral assembly [56]. mCherry is relatively insensitive to pH while the fluorescence of EcpH is fully quenched at pH 6.2, permitting to evidence acidification of the viral environment. The cellular entry was followed by measuring the fluorescence intensities in the green and red channels while perfusing the imaging chamber with solutions of different pH (5.4–8.8). By monitoring the loss of green fluorescence and the sensitivity to media exchange, it was possible to distinguish between population of viruses at the plasma membrane and those in acidic endosomes. The results showed that in almost 80% of the cytoplasmic virions, the EcpH fluorescence was quenched and remained insensitive to pH changes within ~25 s after their cell entry, indicating that the viral complexes rapidly entered into acidic endosomes [50].

In order to evidence the subset of the viruses that enter into the cells by fusion with the plasma membrane, the ICAM1-EcpH labeling was combined with a marker of the fluid phase—imCherry [57]. This combination maximizes the chances to detect the viral fusion at neutral pH, i.e., at the plasma membrane or in the early endosomes. Obtained results showed that the release of the viral content at neutral pH was an extremely rare event. Intriguingly, this release was not affected by fusion inhibitors, suggesting that it resulted from the destabilization of the viral membrane by ICAM1 incorporation rather than from viral fusion. The majority of observed viruses exhibited rapid EcpH quenching followed by directed motion, supporting the fusion from acidic endosomes. Occasionally viral complexes exhibited an ON/OFF blinking, which implies that some virions traffic through different endosomal compartments and are likely recycled at the cell surface. However, the importance of this recycling pathway in HIV-1 infection has not been clarified [57].

Monitoring of the viral content release as a hallmark of fusion was improved by Sood et al. [32], who developed a bifunctional, mCherry-2CL-YFP-Vpr labeling system. This construct enables to follow the viral fusion by the release of intraviral marker mCherry from the viral core labeled by YFP-Vpr [32]. Measuring the kinetics of the fusion in CV-1 derived CD4 expressing cells showed that 50% of fusion events occurred ~13 min after the onset of the infection (by temperature shift from 4 °C to 37 °C). This kinetics was confirmed for both HXB2 Env and VSV-G mediated fusion. Similar fusion timing was observed with HIV-1 viral particles whose Env glycoproteins contained modified sugars (Ac4ManNAz) that were click-labeled by a fluorophore [42].

Similarly, Zongqiang Cui et al. have monitored HIV-1 viral entry in TZM-bl cells by live-cell imaging of infectious HIV-1 virions with encapsulated QD [44]. Single-particle tracking showed that about half of the detected virions first stayed confined to the cell membrane for ~30 min and then moved rapidly into the cell cytoplasm. Interestingly, 82% of HIV-1 virions were recruited in eGFP-clathrin enriched regions of the plasma membrane and then co-localized with transferrin and Rab-5A spots (indicating early endosomes) in the cell cytosol. Treatment by specific inhibitors of clathrin-mediated endocytosis abolished viral internalization. QD, DiD and Rab5A labeling colocalized for about 120 s, and then the QD core marker separated (while the membrane and Rab5A markers stayed together), suggesting endosomal fusion. Of note, that significantly longer time lag (~13 min) between the entry into the endosomes and release was reported by Miyauchi et al. [45].

Overall, fluorescence imaging techniques are a valuable tool for identifying the viral entry pathway. Smart choice of labeling strategies enables the user to distinguish direct fusion with the plasma membrane from endocytosis followed by fusion with endosomal membranes. Several studies confirmed that after the binding to CD4+ receptor and its co-receptors, a fraction of bound virions is internalized via clathrin-dependent endocytosis. The main question that remains is which population (the one located at the plasma membrane or in the endosomes) releases the viral cores to the cytoplasm and thus leads to infection. It has been proposed that cellular entry via endocytosis dominates for HeLa-derived cell lines, while in T-cells, the virus enters by fusion with the plasma membrane. However, this debate is still open and conflicting observations have been reported for CEMss cells. Most likely the balance between the two pathways depends not only on the cell type, but also on additional cellular factors, as discussed in [58].

### 3.3. Factors Enhancing the Viral Entry

HIV-1 has been shown to preferentially infect cells with high metabolic activity [59]. The relationship between cellular glycolytic activity and HIV-1 fusion has been described in an original imaging study by Croomer et al. [60]. Using specific probes for fluorescence lifetime measurements, a higher rate of HIV-1 fusion was shown for CD4+ T lymphoblasts and TZM-bl cells with high ATP:ADP ratio and lactate concentration. Monitoring of doubly labeled (DiD and Gag-GFP) HIV-1 viruses revealed that inhibition of glycolysis blocked viral entry at the plasma membrane at the hemi-fusion stage. A genetically encoded MSS sensor (membrane-bound CFP-YFP FRET-based sensor for membrane tension [61]) was used to show that this down-regulation of cellular metabolism resulted in decreased cholesterol content and plasma membrane tension. Since viral fusion is accompanied by the local change in membrane tension at the site of viral entry, these physical changes of the plasma membrane may be responsible for fusion inhibition in cells with blocked glycolytic activity [60].

In line, an interesting study of the relationship between membrane lipid order and the localization of CD4 and CCR5 receptors in lipid membranes revealed that HIV-1 binds to CD4 receptors in cholesterol rich domains. After this binding, the complex diffuses to the CCR5 co-receptors placed at the boundaries between the ordered and disordered lipid phases. The line tension at these boundaries promotes membrane fusion [62].

## 4. Journey toward the Nucleus

Once in the host cell cytoplasm, the virus begins its journey to the nucleus. Live-cell fluorescence imaging has provided dynamic data on the viral transport through the host cell cytoplasm, while quantitative microscopy studies have shed light on the remodeling of the viral particle during this journey.

The first obstacle at the cell periphery is the cortical actin network. Tracking of QDs-containing HIV-1 in primary macrophages expressing eGFP-Lifeact (F-actin labeling) showed accumulation of F-actin at the site of the viral fusion, followed by generation of a pore-like channel made of cortical actin, allowing the virion to enter the host cell cytoplasm. During channel formation, this site was enriched in α-actinin [54]. In Hela-derived cells, HIV-1 viruses moved along actin filaments at the cell periphery and their movements were blocked by actin polymerization inhibitors, suggesting a role of actin in HIV-1 transport [44].

Several tracking studies using fluorescent HIV-1 models in different cell types have shown that RTCs are transported from the cell periphery towards the nucleus via the microtubule (MT) network. Viral particles exhibit long-range bidirectional displacements resulting in a net movement towards the cell nucleus. Most of them reach the perinuclear area within 1–2 h.p.i. [3,10]. Their velocities vary between ~0.1 µm/s and 1 µm/s and mean square displacement (MSD) plots have shown that they are actively transported [10,44]. All these observations are typical of microtubule-driven transport. In line with this conclusion, the viral complexes co-localize with tubulin, and their movements are inhibited by nocodazole treatment [3].

A recent series of live-cell imaging studies have identified critical host cell factors for this transport. The association of the virus with the MT network has been shown to be mediated by its interactions with CLASP2 (cytoplasmic linker–associated protein 2), a protein that regulates cortical capture and stabilization of MTs [63]. FEZ-1, a kinesin-1 adaptor protein has been shown to link viral capsid to kinesin motors [5]. Two diaphanous (Dia)-related formins Dia1 and Dia2 promoted HIV-1 trafficking to the nucleus by facilitating MT stabilization [11]. Indeed, live-cell imaging and tracking experiments of Vpr-GFP containing HIV-1 viruses revealed that depletion of these proteins resulted in reduced HIV-1 movements to the nucleus as well as a decrease in the number of HIV-1 particles detected within 2 µm of the nucleus at 2 h.p.i. [5,11,63].

In the perinuclear region viral particles exhibited directed movement with reduced velocities (<0.03 µm/s) compared to MT-mediated transport. This type of displacement could be driven by actin. Once at the nuclear envelope, they showed confined and slow movements, reflecting their docking at the nuclear pore complexes (NPCs) [10].

## 5. Cytoplasmic Remodeling of the Viral Core

In addition to dynamic information on the infection time-course, imaging approaches have provided quantitative data about the rearrangements of viral particles. The first study demonstrating intracellular morphology changes in viral complexes was carried out by Stochastic Optical Reconstruction Microscopy (STORM) [64] on HIV-1 viruses immunolabeled against capsid and matrix proteins. Analysis of high resolution (HR) images revealed significant changes in the distribution of both proteins in the viral complexes. After their cytoplasmic entry, the diameters of the protein clusters increased from around 150 nm to 250 nm for the matrix and from 100 nm to 250 nm for the capsid protein, clearly showing a cytoplasmic remodeling of the viral complexes [65]. However, the biological role of this size increase has not been determined.

### 5.1. Cytoplasmic Release of Viral Proteins

Several studies have demonstrated a cytoplasmic release of viral proteins. This release is probably linked to the size reduction needed for the nuclear entry of the virus via NPCs. The size of the mature viral capsid is approximately 120 × 30 nm [66], and the size of PIC has been estimated to be ~56 nm [67]. Since these dimensions exceed the size exclusion limit of nuclear pores ~40 nm [68], the virus must adapt its morphology to pass through NPCs.

Single virion tracking of YFP-Vpr and imCherry containing HIV-1 viruses showed that membrane fusion is rapidly followed by a cytoplasmic loss of the YFP-Vpr signal [20]. Regardless of cell type and envelope glycoproteins (HXB2, VSV-G, ASLV), the YFP-Vpr signal significantly decreased within ~20 min after fusion, and a diffuse nuclear YFP staining appeared at the same time scale. These findings suggest that a fraction of Vpr molecules are released from the cytoplasmic viral complexes and enter the nucleus independently of the viral core. Fluorescence Correlation Spectroscopy (FCS) measurements of the nuclear YFP-Vpr showed two populations of diffusing molecules indicating that in the nucleus Vpr binds to larger complexes [20], but the biological role of this binding remains unclear.

**Figure 3:**
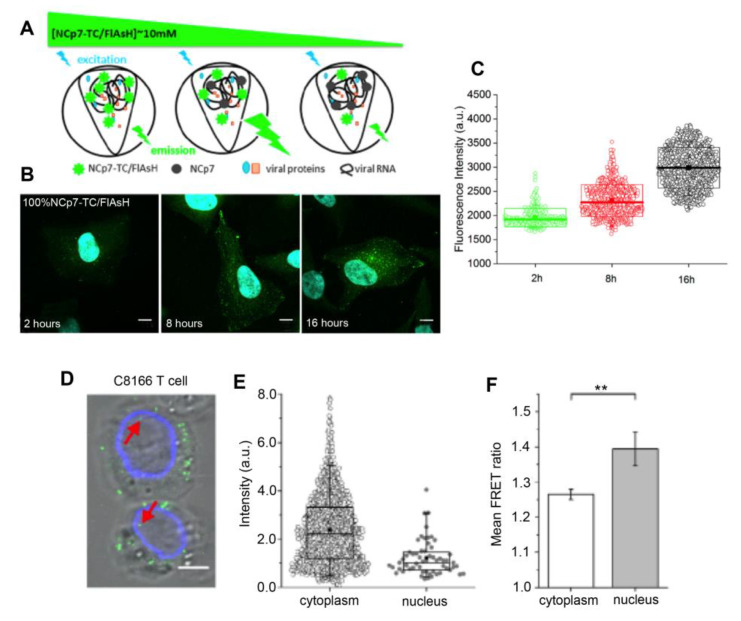
Fluorescence-based monitoring of the cytoplasmic release of viral proteins: (**A**) Principle of FlAsH fluorescence quenching at high concentrations. A decrease in the concentration of labeled NCp7 first results in an increase in fluorescence emission due to the reduction in quenching, followed by a decrease when the quenching effect is no longer present and the number of NCp7 molecules decreases further in the complexes. (**B**) Confocal images of HeLa cells infected by NCp7-TC/FlAsH-containing VSV-G pseudotyped HIV-1 particles at 2, 8, and 16 h.p.i., Scale bar: 10µm (**C**) Fluorescence intensity of individual cytoplasmic viral complexes detected at 2, 8, and 16 h.p.i. The fluorescence increase reflects the loss of quenching during the release of NCp7-TC/FlAsH molecules. (The box-plot represents SD values, the line and the square represent the median and the mean value, respectively (adapted from [22], copyright 2019, Springer Nature)). (**D**) Confocal images of C18166 T cells infected by VSV-G pseudotyped HIV-1 containing IN-GFP (green spots), lamin immunostaining (blue); red arrows indicate viral particles located in cell nuclei (**E**) Intensity of fluorescent spots detected in the cytoplasm and the nucleus (box-plot whiskers represent 5th and 95th percentile, the line and the square depict median and mean value, respectively) (**F**) Mean FRET ratio of HIV-1 pseudoviruses containing IN-mTFP and IN-mVenus in the cytoplasm and the nucleus, ** *p*-value < 0.05 (reproduced with permission from the Ref. [21] Copyright 2016, Springer Nature).

Likewise, a cytoplasmic release of NCp7 during the early stages of infection has been shown in a fluorescence quenching-based microscopy study (Figure 3A–C) [22]. The fluorescent signal of TC/FlAsH labeled NCp7 in VSV-G pseudotyped HIV-1 was monitored during the first 8 h and 16 h of infection in HeLa cells. Since fluorescein-based dyes show self-quenching at high concentrations, a decrease of NCp7-TC/FlAsH concentration inside the viral cores resulted in increased fluorescence emission of individual pseudoviruses (Figure 3A). Analysis of the fluorescence intensity of the cytoplasmic NCp7-TC/FlAsH spots revealed an increase in fluorescence at 8 h.p.i. and 16 h.p.i. compared to 2 h.p.i. (Figure 3B,C). By comparing the FlAsH signal with a calibration curve, the results revealed that at 8 h.p.i., roughly 40% of NCp7-TC/FlAsH molecules are released from the viral cores and that up to 70% are released at 16 h.p.i. This increase was more pronounced near the nuclear periphery and was suppressed by RT inhibition, indicating that the release of NCp7 protein might be related to viral transport and reverse transcription [22].

Moreover, photoactivation localization microscopy (PALM) [69] and a quantitative FRET-based microscopy study [21] have shown the reduction in the size of IN-containing complexes, and a decrease in the number of IN molecules between the cytoplasm and the nucleus. Since the size of HIV-1 virions is below the resolution limit of classical optical microscopes, the study of virus morphology changes requires HR microscopy. PALM [70] imaging of FlAsH-labeled IN-TC in VSV-G pseudotyped HIV-1 and statistical analysis of individual IN-containing clusters was used to distinguish the size and shape of mature and immature viral particles. This analysis further showed a significantly decreased size for the nuclear IN-TC/FlAsH spots (FWHM ~41 nm) compared to the cytoplasmic spots (FWHM~98 nm). Of note, it was not possible to determine whether the nuclear IN clusters belong to pre-integration complexes (PICs) or correspond to IN molecules released from the viral cores [69].

The reduction in the IN content of nuclear PICs has also been demonstrated in a quantitative FRET-based study [21]. First, the fluorescence intensity of individual IN-GFP containing HIV-1 viruses was measured by confocal imaging in HeLa P4, T cell line (C8166), and primary CD4+T cells at 24 h.p.i. A two-fold decrease in nuclear GFP signal was observed compared to cytoplasmic spots (Figure 3D,E). Next, HIV-1 pseudoviruses containing IN-mTurquoiseFP and IN-mVenus were used for FRET intensity measurements after acceptor photobleaching. A higher FRET ratio was observed in nuclear PICs compared to cytoplasmic complexes (Figure 3F), witnessing changes in the stoichiometry or the oligomerization state of IN molecules within the viral core. Both effects were independent of Transportin 3 (TNPO3), the cellular factor that assists nuclear import of HIV-1 [71]. Depletion of LEDGF/p75, which guides the PIC to its integration site [72] did not affect the IN content but prevented the nuclear FRET ratio increase indicating that the LEDGF/p75 binding affects the oligomerization state of IN. Overall, these data clearly demonstrated the release of IN molecules and changes of IN oligomerization upon nuclear entry of the PICs [21].

### 5.2. Reverse Transcription and Proviral DNA Imaging

One of the most important remodeling steps during the early phase of HIV-1 infection is linked to reverse transcription. Many incoming virions fail to synthesize vDNA and turn into infectious PICs. Therefore, the ability to identify viruses that go through this stage helps to uncover the key factors leading to effective infection. The first imaging of the vDNA genome was carried out by Mc Donald et al. [3], who labeled the vDNA of GFP-Vpr containing HIV-1 viruses by microinjecting fluorescent nucleotides (AlexaFluor-594-UTP) into infected cells. These nucleotides were incorporated into vDNA during reverse transcription. The images showed an accumulation of viral genomes at the nuclear periphery, near the microtubule organizing center (MTOC) [3].

Later, the staining technique was improved by using cell-permeant nucleotides bearing an alkyne group (5ethynyl-2-deoxyuridine) that are labeled by click reaction with fluorescent azides [40,41]. Using this approach, Peng et al. [41] observed the presence of vDNA in infected TZM-bl cells already 2 h.p.i. Their number increased to five DNA-containing viral particles/cell at 4 h.p.i. and remained constant until 6 h.p.i. Cytoplasmic PICs co-localized with IN-GFP and immunolabeled CA and NCp7 proteins. In primary macrophages, detection of vDNA was less frequent due to inhibition of reverse transcription by the cellular HIV-1 restriction factor sterile alpha motif and histidine/aspartic acid domain-containing protein 1 (SAMDH1) [41]. SAMDH1 depletion increased the number of PICs detected at 24 h.p.i. up to 6.4 per cell in the cytoplasm and 4.4 per cell in the nucleus [40]. The use of the MICDDRP approach to quantify the disappearance of vRNA and the appearance of vDNA signals at different time points confirmed that vDNA already appears at 2 h.p.i. and that reverse transcription is completed by ~10 h.p.i. [39].

Reverse transcription is believed to occur in the host cell cytoplasm, but recent reports indicate that vDNA could be synthesized in the cell nucleus. Time of addition assays with nevirapine (NVP), an inhibitor of reverse transcription showed that escape from its inhibition occurred ~4h after nuclear entry, indicating that vDNA is synthesized in the nucleus [18,73].

Campbell’s team [74] has developed an elegant inducible system for blocking nuclear pores using cells stably expressing Nup62 (NPC protein) fused to a drug inducible dimerization domain B and two copies of eGFP, Nup62-DmrB-2eGFP. This system enables to block active nuclear transport by a rapamycin analogue homodimerization drug (HD). Addition of HD and NVP at different times of infection confirmed that nuclear import occurs in less than 4 h.p.i. and that the virus remains sensitive to RT inhibition several hours after its escape from NPC blockade. Importantly the presence of NVP enhanced the sensitivity to nuclear import inhibition. These results indicate that the reverse transcription is completed in the cell nucleus, but it has most likely started in the cytoplasm. In addition, the onset of RT in the cytoplasm may be necessary for efficient nuclear import [74].

The analysis of vDNA in infected monocyte-derived macrophages (MDMs) by EdU labeling confirmed these results. Bejarano et al. measured higher intensities in nuclear spots compared to complexes located near the nuclear envelope [75], and Francis et al. observed an increase of EdU labeling of nuclear viral complexes with time. Moreover, this increase was abolished by NVP treatment [73].

Finally, several studies have shown that reverse transcription is not required for nuclear entry [12,13,17,18,37,73,76]. Altogether these data clearly indicate that the vDNA is at least partly synthesized in the cell nucleus and hence redefine the conventional order of the key steps of HIV-1 infection.

## 6. Uncoating

The orchestrated disassembly of the viral capsid has probably been the most controversial step in the HIV-1 life cycle and despite extensive investigations, the process of HIV-1 uncoating remains unclear (reviewed in [8,9]). The mature viral capsid is a cone-shaped envelope in which ~1500 CA proteins are organized in a fullerene lattice composed of CA hexamers and a few pentamers [77,78]. This shell protects the viral genome against cellular defense mechanisms during cytoplasmic transport. Intact viral capsids are too large to cross the nuclear pores, it has therefore been classically accepted that uncoating takes place in the host cell cytoplasm. A series of imaging-based studies have shown that uncoating is linked to reverse transcription and viral transport via microtubules. However, since CA proteins play a crucial role in nuclear import and targeting of the PIC to its integration site, it is obvious that at least some CA proteins enter the nucleus of the host cell.

### 6.1. Capsid Opening

Mamede et al. [15] reported a live-cell imaging study of the kinetics of capsid opening by monitoring the release of internal GFP marker from viral cores. Different cell types (CHO, CD4+ T cells, and MDMs) were infected by VSV-G pseudotyped HIV-1-iGFP-NL4–3 viruses and imaged. Regardless of cell type, the iGFP was released in two steps. First fluorescence decrease was observed after 30 min of cell engagement and reflected the iGFP release during fusion. After this step, majority of viruses still emitted fluorescence signal, due to a subset of iGFP molecules present in the capsid core. Second loss of iGFP occurred, dependent on cell line, between 17 min and 25 min after viral fusion. This second release correlated with substantial, but not complete loss of CA staining (immunolabeled or CA-TC/ReAsH) [15]. Moreover, by imaging cells infected by a single virus, the authors related this early capsid breach to productive infection, identified by Gag-eGFP expression several hours later. Capsid opening was delayed by NVP and CA N74D mutation, but not by inhibition of the RNase-H activity of the reverse transcriptase (resulting in a blockade at the first-strand transfer step). These observations support the hypothesis that capsid disassembly starts early after the cellular entry of the virus and is triggered by the onset of reverse transcription [79,80].

A similar timing of capsid opening was observed by Xu et al. [16], who produced HIV-1 containing vRNA labeled with 5-ethynyl uridine (EU). The loss of capsid integrity was monitored in TZM-bl cells by the efficiency of EU labeling reflecting the accessibility of the EU substrate for the click reaction. In agreement with Mamede et al., the number of EU spots increased between 15 min and 45 min p.i. Fluorescence images of Vpr-GFP containing HIV-1 immunolabeled against p24, showed a significant decrease of RNA co-localization with CA and Vpr signals at 1 h.p.i., reflecting their dissociation from the viral complexes. In contrast, the anti-NC immunostaining remained co-localized with RNA puncta, in line with the role of NC during the reverse transcription [16]. The RNA accessibility kinetics was modified by capsid mutations altering the stability of the cone, however, contrary to Mamede et al. it was not affected by RT inhibition.

In conclusion, using two different imaging approaches, these studies showed, that the integrity of the capsid is rapidly compromised (~30 min) after viral cell entry and that it is likely linked to the onset of reverse transcription.

### 6.2. Uncoating and Cytoskeleton

In addition to RT, HIV-1 uncoating is related to viral transport and nuclear entry. These connections have been highlighted by the important role played by cytoskeleton-related proteins. Diaphanous (Dia) related formins (DFRs) are proteins which coordinate cytoskeletal remodeling by controlling actin nucleation and microtubule stabilization. This stabilization promotes HIV-1 transport from the cell periphery to the nucleus. Two members of the DFR family, Dia1 and Dia2 have been shown to be also involved in HIV-1 uncoating. By measuring the CA content of viral particles in CHME3 cells, the authors showed that the depletion of Dia1 and Dia2 inhibited the loss of the capsid. Interestingly, the role of both DFRs in viral uncoating was independent of their ability to stabilize the MT network [11].

Likewise, a MT-related Kinesin-1 (KIF5B) motor plays a role in both processes. KIF5B binds to the viral capsid and mediates its transport to the NE, where the complex docks at the NPC via capsid binding to nucleoporin 358 (Nup358). Analysis of immunolabeled CA proteins and Nup358 cellular distributions in HeLa cells and MDMs indicated that HIV-1 infection causes a KIF5B-dependent displacement of Nup358 from the nuclear envelope (NE) to the cytoplasm. In addition, at the cell periphery, Nup358 co-localizes with the CA staining. These observations raise the hypothesis that at the nuclear pore level, the KIF5B-bound capsid docks on Nup358 and is mechanically disrupted by the forces exerted by kinesin. CA fragments as well as Nup358 are then transported by KIF5B to the “+” end of MTs and the PIC most likely enters into the cell nucleus [4].

More recently, HIV-1 induced relocation from the NPCs was shown also for Nup62 [74]. Similar kinesin induced alterations of NPCs were reported for adenoviral infection, where they promote the viral entry by increasing the permeability of nuclear pores [81]. No such effect was observed for HIV-1, nevertheless it would be of interest to evaluate the integrity of the nuclear barrier during the HIV-1 infection, since NPCs disruption would enable the nuclear entry of a large part of capsid shells or even intact capsids, as described in Section 6.5.

### 6.3. Uncoating at the Nuclear Pore

In support of the current understanding of the time course of capsid disassembly, Zila et al. [19] have proposed an interesting interpretation of the early loss of capsid fluorescence signal. This group studied the fate of the capsid by super-resolution (STED) microscopy imaging of immunolabeled CA in viruses containing IN-GFP and membrane-labeled by mCLING (a membrane-binding fluorophore that stably attaches to the plasma and endosomal membranes [82]). By comparing the CA content of HIV-1 particles that entered CD4+ T cells with unfused viruses, the authors showed that about 50% of the CA content is lost shortly after fusion. Authors hypothesized that this capsid loss reflects the release of free CA molecules present in the viral core and not the disassembly of the capsid shell. Indeed, more than 5000 Gag polyproteins are incorporated into each viral particle during the budding, but only 1500 form the capsid lattice. Therefore, after maturation, a fraction of capsid molecules remains trapped within the viral core and might be released during the fusion, while the capsid shell disassembly is completed at a later stage. In line with this hypothesis, the remaining CA signal was lost at the NE within 70 nm from the NPCs [19].

This late uncoating model has been suggested also by PALM imaging of IN-TC/FlAsH labeled HIV-1 pseudovirus in HeLa P4 cells. Statistical morphology analysis of cytoplasmic IN-TC/FlAsH clusters showed that they exhibit a conical shape with average dimensions of ~108 nm × 75 nm similar to mature viral particles [69]. These values are in good agreement with the dimensions of viral capsids determined by TEM microscopy [66] and support the hypothesis that almost complete capsid shells are present in the cell cytoplasm.

**Figure 4:**
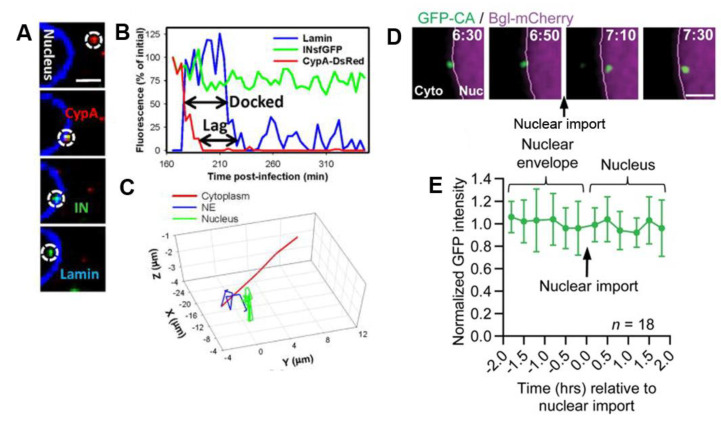
Two conflicting examples of uncoating studies based on live-cell imaging of the loss of capsid labeling. (**A**) Imaging of uncoating at the nuclear pore by time lapse imaging of HeLa-derived cells expressing EBFP2-Lamin and infected with INsfGFP/CypA-DsRed-labeled HIV-1 pseudoviruses. The red signal (CypA-DsRed) is lost upon nuclear entry. (**B**) Fluorescence intensity time trace and (**C**) trajectory of a viral particle revealing the capsid loss during docking at the nuclear envelope characterized by confined movements (reproduced with permission from [13], copyright 2018, Cell Press). (**D**) Time lapse images of CA-eGFP labeled viral particles in HeLa cells expressing BglG-mCherry (located in the cell nucleus) from 6.5 h.p.i. to 7.5 h.p.i. (scale bar 2 µm) (**E**) The normalized mean intensity time trace of CA-eGFP from the viral particle indicate that most CA-eGFP molecules remain associated with the viral core upon nuclear entry (reproduced with permission from [18], copyright 2020, PNAS).

The importance of capsid in relationship to successful nuclear entry leading to productive infection has been nicely described in a series of live-cell dynamic studies published by Francis et al. [13,17,74,76]. This group took advantage of the specific binding of cyclophilin A (CypA) to the HIV-1 capsid and produced HIV-1 pseudoviruses labeled by CypA fused to DsRed (CypA-DsRed). IN-super-folderGFP (INsfGFP) was used as a viral core label. Dual-color live-cell imaging of the infection revealed that in the majority of viral particles, CypA-DsRed fluorescence was lost approximately 20 min after fusion and was followed by loss of IN-sfGFP signal due to degradation of the viral core by cellular proteases. For the remaining viral complexes (<5%), the loss of CypA-DsRed signal occurred several hours later, upon docking to the NE. Unlike cytoplasmic uncoating, late capsid loss was not affected by inhibition of reverse transcription. Examples of confocal images and corresponding time traces of the loss of CypDs-Red signal at the NE (revealed by lamin staining) are shown in Figure 4A,B. Viral complexes (Figure 4C) undergo directed cytoplasmic movement followed by docking and confined displacement in the cell nucleus. After uncoating, viral complexes remained docked at the nuclear periphery for an additional ~16 min before entering the nuclear compartment where the sfGFP signal was lost, probably due to PIC disassembly after integration. Several hours later, infected cells expressed GFP as a reporter for infection. Interestingly, 30% of intranuclear sfIN-GFP spots were positive for CypA-DsRed signal, indicating that a part of capsid enters into the nucleus [13,17].

In terminally differentiated human MDMs, 80% of the cytoplasmic viral complexes retained the CypA-DsRed signal for several hours. Early uncoating was stimulated by SAMHD1 depletion, which promotes reverse transcription, confirming that the latter favors early capsid disassembly. Importantly, as in HeLa cells, the viral complexes, which entered the cell nucleus lost their capsid staining at the NE [73].

Altogether, these data suggest that early cytoplasmic uncoating leads to the proteasomal degradation of the virus, and its extent depends on host cell factors. On the contrary, late uncoating scenario ensures protection of the viral genome during the cytoplasmic transport to the NE and viral docking at the NPCs. The viral capsid disassembly at the nuclear pore leads to nuclear entry and infection regardless of cell type.

### 6.4. Capsid in the Nucleus

A growing body of evidence indicates that the capsid shell might be disassembled at the nuclear pore level and that a fraction of more or less arranged CA proteins enter the cell nucleus. High resolution structured illumination microscopy (SIM) showed the presence of immunolabeled CA associated with nuclear IN-GFP containing viral complexes in CHO cells at 6 h.p.i. [29]. Quantification of the CA signal showed a five-fold decrease in intensity between the nuclear and cytoplasmic complexes, confirming late uncoating at the nuclear pores. Similarly, Peng et al. and Chin et al. showed detectable CA signals that co-localized with vDNA in the nucleus of infected HeLa and MDM cells [38,41].

Intriguing observations have been made by imaging pNL4.3 derived HIV-1 viruses in which a fraction of the CA proteins has been fused to eGFP (CA-eGFP) [28]. CA-eGFP labeled viruses contained an average of 2 eGFP molecules, located most probably in the viral core. Along with the Vpr-mediated trans-incorporation of IN-mCherry, the doubly labeled viral particles were used to follow intracellular capsid trafficking. First of all, observing viral complexes containing only one of the labels, the authors demonstrated different distributions in the cytoplasm, at the level of the nuclear envelope and in the cell nucleus. The intensity of the nuclear IN-mCherry signal dropped significantly relative to the cytoplasm in line with the previously reported IN release prior to nuclear entry [21]. CA-eGFP nuclear spots showed intensity comparable to that of the cytosolic population, but a decrease of CA-eGFP labeling was observed for 15% of the brightest cytosolic spots. Since CA-eGFP reside rather in the viral core and only exceptionally in the capsid shell, these results indicate that part of the CA molecules is lost at the NE, while remaining CA molecules enter the nucleus, in agreement with the late uncoating model. Analysis of the doubly labeled particles confirmed the differences in cell distribution since 20% of the IN-mCherry positive spots in the cytoplasm also contained CA-eGFP, but this fraction dropped to 15% at the NE level and 7% in the nucleus. The fact that only 3% of nuclear CA-eGFP complexes also contained IN-mCherry indicates that CA separates from the viral core at the NE and enters the nucleus independently of the PICs. Moreover, integration inhibition by raltegravir increased the number of IN-mCherry complexes in the nucleus while no effect was observed for CA-eGFP spots, confirming that nuclear CA-eGFP proteins are not part of PICs or that they dissociate from the viral complexes before integration [28]. This article raised thus, the interesting hypothesis of independent entry of capsid proteins into the cell nucleus.

### 6.5. Uncoating in the Nucleus

In their recent papers, Krausslich’s group analyzed the intensity of CA immunostaining in infected MDMs. Obtained results showed no or only moderate decrease in CA signal between viral complexes located in the cytoplasm and nucleus. Moreover, nuclear CA staining was sensitive to PF74 treatment (capsid destabilizing drug) and cleavage and polyadenylation factor 6 (CPSF6) depletion (facilitating the nuclear import), both binding to CA hexameric lattice. These observations suggest the presence of a large part of the CA shell in the cell nucleus [19,75].

In agreement, the report by Burdick et al. proposed a new HIV-1 uncoating scenario in which the capsid shell remains intact until the integration step [18]. Doubly labeled GFP-CA and A3F-RRvT (red-red vine Tomato) HIV-1 viruses were used to infect HeLa, CEM-SS T-cells and TPH-1 derived macrophages and the fluorescence intensities of the two labels were analyzed with time. Only a slight loss of GFP-CA signal was observed in the cell cytoplasm. Interestingly, the signal remained constant when the viral complexes crossed the NE (Figure 4D,E) but dropped drastically in the nucleus at 10.5 h.p.i. The viral transcription sites (visualized by BglG technology) appeared 8.4 h after the loss of the capsid and the expression of the GFP reporter was detected 3 h later. Interestingly, time of addition assays using NVP, raltegravir, and PF74 revealed that loss of sensitivity to PF74 occurred 3 h after the NVP escape and 1 h before escape from RAL inhibition. Since the mean time measured for nuclear entry was 4.4 h.p.i., the authors conclude that capsid disassembles in the nucleus after the RT step.

Altogether, the reported data suggest that almost intact viral capsids enter the nuclear compartment and that uncoating occurs inside the nucleus after reverse transcription. It should be noted that in order to preserve the infectivity of the virus, only a fraction of CA molecules was fused to GFP and these GFP-CA proteins were probably incorporated into the viral core rather than into the capsid shell as detailed in [28]. Therefore, it is questionable if this model is suitable to faithfully monitor the uncoating step. It remains also to determine the mechanism of nuclear import of the capsid shell whose dimensions exceed the exclusion limit of nuclear pores. The authors hypothesized that CA interactions with cellular factors (CPSF6) could modify the structure of the viral complex or of the NPCs to facilitate the nuclear import of the capsid shell [18].

In recent years, imaging studies have provided a wealth of data on capsid disassembly, but this process remains still controversial. The understanding of HIV-1 uncoating is likely compromised by discrepancies resulting from differences in the CA labeling strategies and cell models used (Table 2). Immunostaining does not interfere with the infection process, but its readout depends on antibodies and the accessibility of the epitopes. Approaches based on labeling via an interacting partner, such as Cyp-DsRed, are well suited for live-cell imaging, but the result of this indirect labeling might be biased by CypA-CA binding. Recently reported GFP-CA and CA-eGFP fusions only label a small fraction of CA molecules, and their incorporation into the fullerene lattice is questionable.

The early uncoating model was the preferred model for several years, but recent studies have shown that although the integrity of the capsid cone may be compromised soon after cell entry, disassembly of the protective shell most likely occurs at the nuclear pores and part of the capsid structure enters the cell nucleus. The relationship between uncoating and the onset of reverse transcription, the cytoplasmic transport, and the nuclear entry has been demonstrated and the role of CA in nuclear events has become increasingly evident. The latest proposed model of intact viral capsids in the cell nucleus is of interest and will surely be at the center of future investigations.

## 7. Nuclear Entry

HIV-1 delivers its genome into the nuclei of non-dividing cells by hijacking the cellular machinery and enters into the cell nucleus by active import via NPCs. Nuclear entry occurs relatively rapidly. Live-imaging studies have shown that in HeLa cells the first viruses reach the nuclear compartment already 1–2 h.p.i. with an average nuclear import time of about 4.3 h.p.i. [10,12,13,39,41].

Nuclear entry is a highly selective process. Indeed, the majority of intracellular virions fail to enter the nucleus and remain in the cytoplasm as non-infectious end products. Time-lapse imaging studies describing the kinetics of the early stages of infection have revealed that successful nuclear entry of the virus requires appropriate docking of the viral core on the cytoplasmic side of the NPCs [12,13]. By following A3F-YFP or IN-YFP labeled VSV-G pseudotyped HIV-1 viruses in HeLa cells, Burdick et al. [12] showed that the majority of the viral particles only briefly tickle the nuclear envelope (NE) before diffusing back to the cytoplasm. Only a small fraction remained attached to the cytoplasmic side of the NE for more than 20 min (mean residence time = 1.5 ± 1.6 h) and entered the nucleus.

This lasting attachment to nuclear pores called “docking” was also reported by Francis et al., who showed that the virus uncoats during this step [13]. Viral docking is dependent on capsid binding to Nup358 at the cytoplasmic side of NPCs and is related to capsid stability. Indeed, facilitating viral uncoating by inhibition of CypA-CA interactions, decreased the time the viruses spent in cell cytoplasm and at the NE, however, it did not affect the efficacy of nuclear import [12]. Additional cellular factors such as CPSF6 and Nup153 are possibly involved, since treatment with PF74, which interferes with their binding to viral capsid, displaced the docked complexes from the NE and reduced the nuclear import [13]. These dynamic studies have clearly evidenced that the docking and uncoating at the NPCs are essential for the nuclear import of the viral genome.

HIV-1 transport through the nuclear pores remains poorly understood, although Nuclear Localization Sequences (NLS) have been identified on most PIC components and several viral proteins have been shown to interact with cellular importins and nucleoporins [83,84,85,86]. Among other cellular factors, Nup 153 and TNPO3 have been shown to participate in HIV-1 nuclear entry through their interactions with the viral capsid. Nup 153 is a component of the NPC basket. Its binding to the cyclophilin binding pocket of the viral capsid promotes viral uncoating, translocation of PIC through the nuclear pore and guides the selection of the integration site. Depletion of Nup153 considerably reduces viral infectivity [84,85,87,88]. TNPO3 is a member of the importin-β family, which is crucial for viral infectivity. It participates in the nuclear entry via integrase and capsid binding or indirectly by modifying the intracellular localization of CPSF6. Its depletion reduces integration but not LTR formation [71,89,90]. A recent study identified Transportin-1 (TRP-1) as a key player in HIV-1 uncoating and nuclear import [91]. The authors show a co-localization of TRP-1 and CA near the NE. Their interaction was further confirmed by a proximity ligation assay and the NLS sequence responsible for TRP-1 binding was identified. Depletion of TRP-1 abolished the infection by preventing both nuclear import and the uncoating. Interestingly, TRP-1 depletion did not affect the cytoplasmic trafficking of viral particles, but impacted their capacity to properly dock at the NE and enter the nucleus [91]. These results indicate the crucial role of capsid binding to the cellular nuclear import machinery for nuclear entry of HIV-1.

## 8. Inside the Nucleus

Imaging of nuclear PIC has been technically challenging for a long time. In early tracking studies with Vpr-FP or IN-TC/FlAsH labeled HIV-1, the fluorescent spots disappeared before or shortly after nuclear entry, possibly due to the release of the fluorescent labels during rearrangements of viral complexes linked to nuclear import [3,10]. Better adapted labeling strategies such as A3F-YFP or Vpr-mediated IN-GFP incorporation, have allowed to track the intra-nuclear PICs until the integration site [30,37]. As mentioned earlier, viral particles can be detected in the nucleus from 2–3 h.p.i. with a maximum at around 6–10 h.p.i.

### 8.1. Intranuclear Trafficking towards the Site of Integration

**Figure 5:**
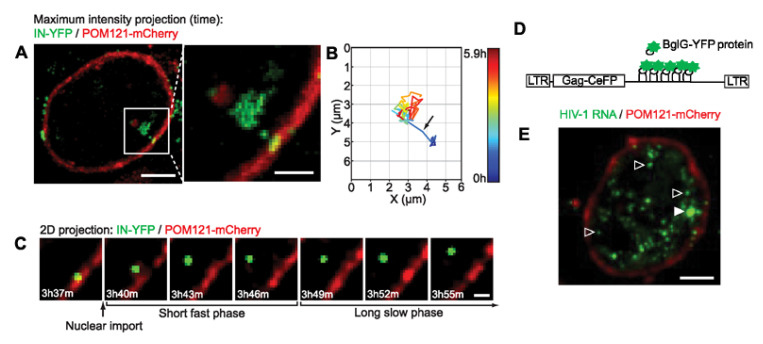
Monitoring of HIV-1 in the nucleus. (**A**) 2D projection of the 3D tracking of an IN-YFP labeled HIV-1 complex in HeLa cells expressing POM121-mCherry as an NPC marker. After a long initial docking at the nuclear envelope, the complex enters the nucleus. Scale bar: 5 μm. (**B**) Trajectory of the imaged particle (**C**) Representative images of nuclear entry followed by a phase of rapid movements and a second phase of reduced mobility. Scale bars: 2 μm. (**D**) Schematic of the method of HIV-1 pseudogenome labeling (**E**) Confocal image of the sites of BglG-YFP accumulation in the nucleus of an infected HeLa cell. The black arrows show the viral RNA and the white arrow shows the transcription site. Scale bar: 5 µm. (Reproduced with permission from the Ref. [12] Copyright 2017, PLOS).

Tracking of A3F-YFP-labeled viral particles in the nucleus of HeLa cells showed that they initially diffused rapidly, but within a few minutes, their diffusion was confined with velocities lower than 0.005 µm/s [10,12,13]. Maximum intensity projection and an example of reconstructed trajectory of a nuclear viral complex are shown in Figure 5A,B, respectively. Figure 5C shows time lapse images of a viral particle entering the nuclear compartment. The diffusion coefficients (D) measured for this slow phase range between 0.3–0.6 × 10^−4^ µm^2^/s [12]. These values are comparable to the diffusion coefficient D measured for the nascent RNA transcripts (TS) by BlgG-YFP (D = 0.6 × 10^−4^ µm^2^/s) (Figure 5D,E white arrow). These findings support the hypothesis that once in the nucleus, viral complexes bind quickly to cell chromatin [12].

Several studies of the intranuclear distribution of HIV-1 have shown that viral complexes localize near their entry point at distances between 0.4–2.5 µm from the nuclear periphery [12,13,30,37]. Similarly, vDNA (integrated or not) imaging has confirmed this preferential location [38,46]. Marini et al. established a list of HIV-1 recurrent integration genes (RIGs), which correspond to a subset of gene integration sites, the most frequently targeted by the virus [92]. In CD4+ T cells, 63% of RIGs were found localized within 1 µm of the NE. In agreement, 73% of HIV-1 nuclear genomes labeled by FISH were concentrated in this zone. This localization was lost when integration was impaired by IN mutations or raltegravir treatment.

### 8.2. Factors Determining the Choice of the Integration Site

The choice of the site of HIV-1 integration has been found to be linked to the nuclear architecture. Correlation of A3F-YFP-labeled viral complexes with a marker of transcriptionally silenced heterochromatin (K9H3me3) revealed that PICs are excluded from regions of condensed chromatin, [30,93].

Lelek et al. have shown that HIV-1 integration may result from the organization of chromatin near the NE which is, at least in part, governed by NPCs [94]. Depletion of Tpr, a nucleoporin that forms filaments protruding into the nuclear region, drastically reduced viral integration. Dual-color STORM imaging showed that Tpr depletion caused a reduction in actively transcribed heterochromatin (labeled by H3K36me3 marker) at distances between 100 nm and 500 nm from NPCs. This modification of the chromatin topology underneath the NPCs, leading to the removal of actively transcribed genes that are preferential HIV-1 integration sites, could be responsible for the decrease in viral replication in Tpr-depleted cells.

In addition, intranuclear trafficking of HIV-1 is driven by viral interactions with cellular factors. Tpr and Nup153 have been shown to promote the peripheral localization of LEDFG/p75, a cellular factor that drives the PIC to actively transcribed chromatin [94]. In agreement, depletion of Nup153 and LEDGF/p75 abolish the peripheral localization of vDNA in CD4+ T nuclei [92]. Of note, the dependence of the peripheral localization of vDNA on LEDGF/p75 has not been confirmed in HeLa cells [93].

Another important cellular determinant for HIV-1 in the nucleus is CPSF6 [38,46]. CPSF6 is a viral restriction factor that binds to CA lattice in the cell cytoplasm and blocks the infection by enhancing CA stability [95,96]. Intriguingly in the cell nucleus CA-CPSF6 interactions favors the infection by facilitating the nuclear import and PIC targeting to the integration site [18,19,38,73,75,76].

Bejarano et al. demonstrated the recruitment of CPSF6 in nuclear HIV-1 complexes in MDM cells infected with IN-eGFP containing HIV-1. Upon infection, CPSF6 formed clusters that co-localized with CA, vDNA, and IN-eGFP spots in the cell nucleus, while no CPSF6 was detected in cytoplasmic viral complexes. Interestingly, 80% of the CPSF6-positive nuclear spots also contained a detectable LEDGF/p75 signal. STED imaging revealed that CPSF6 depletion led to blockade of CA-positive viral complexes at the nuclear pores, where they partially co-localized with Nup153 in the NPC basket [75].

A similar accumulation of CA-containing viral complexes at the NE upon alteration of CA-CPSF6 binding has been reported by Zila et al. in T cells [19]. In contrast, Burdick et al. observed in HeLa cells that perturbation of CA-CPSF6 binding led to capsid uncoating at the NE level while the viral core entered into the nucleus. Regardless of whether the capsid shell disassembles at the NE or later, all reports indicate that CPSF6 is recruited by the viral capsid at the nuclear pores and their interactions facilitate the nuclear import of the virus [75].

The role of CPSF6-CA interactions in targeting viral complexes to their integration sites has been further described in live-cell imaging study by Francis et al. [76]. Tracking of HIV-1 labeled by IN-mNeonGreen and CypA-DsRed in a variety of cell types showed that, after nuclear entry, several viral complexes fuse to form clusters in chromatin region enriched with actively transcribed genes, called nuclear speckles (NS). Viral clusters are located at the site of both, integration and transcription. Alteration of CA-CPSF6 binding by PF74 or mutations in one of the partners resulted in delocalization of the viral clusters out of NS and their disappearance. Consistent with these observations, analysis of HIV-1 integration sites showed that inhibition of CA-CPSF6 interaction abolished the preferential integration of vDNA into NS-associated genomic domains, SPADs. Moreover, depletion of CPSF6 resulted in a decrease in RIGs associated with SPADs. Altogether, these results identify SPADs as the preferential sites for viral integration and the role of CA-CPSF6 interactions in targeting HIV-1 to these genomic regions [73,76].

It should be noted that, despite the role of CPSF6 in the nuclear entry and integration of HIV-1, its depletion or the inhibition of CA-CPSF6 binding has no or only a moderate effect on viral infectivity [19,75,96], likely because the virus is blocked in the nuclear basket, but still integrates into chromatin in the immediate vicinity of the NPCs [18,19,38].

In conclusion, imaging studies have shown that after nuclear entry, HIV-1 complexes bind rapidly to transcriptionally active chromatin near the NE. The interplay between chromatin topology and cellular factors (LEDGF/p75, Nup153) is likely crucial for the selection of the integration site. Interactions of CPSF6 with the viral capsid play a key role in nuclear import and targeting of PIC to its integration site.

## 9. Conclusions and Perspectives

Advances in fluorescence microscopy approaches and labeling strategies have led to the emergence of numerous imaging studies of the HIV-1 life cycle. Compared to biochemical and molecular biology methods that provide information about bulk virus population, imaging approaches have the advantage of monitoring individual viruses and identifying viral particles that trigger productive infection. From the initial imaging of the distribution of HIV-1 in infected cells, various strategies were rapidly developed to enrich obtained data by quantitative information. Dynamic studies have provided valuable details on the kinetics of the different steps of a viral infection. Additionally, a smart combination of labeling strategies permitted to monitor the location and timing of viral disassembly and uncoating, highlighting the release of viral proteins during the remodeling of viral complexes. Single virus tracking helped identifying, in a large population of viral particles initially present, the small fraction that triggers productive infection. It became thus possible to describe the key infection pathway, even though it is taken only by a minority of viral complexes.

Despite considerable efforts, several steps of the early stages of the viral life cycle remain unclear. The conflicting findings of the latest uncoating studies will surely inspire further research. The size and integrity of capsid structures in the cell nucleus and their role in integration remain to be determined. Because of their high spatial resolution, HR microscopy and combination of photonic and electronic microscopy (CLEM) will be particularly suitable for these studies. Similarly, the mechanism of the nuclear entry of HIV-1 remains unclear. The role of the capsid and several cellular factors has become evident, but the mechanism by which the large viral complexes pass through the narrow central channel of NPCs remains unclear. Innovative HR microscopy techniques and quantitative image analysis will enable to analyze the morphology of the viral complexes near the nuclear pores and to identify the main remodeling steps leading to the nuclear entry. The fact that HIV-1 displaces some Nups from the NPCs indicates that in order to access the nucleoplasm, the virus may alter their permeability. The mechanism of viral nuclear entry represents an exciting challenge for future investigations.

As the size of HIV-1 is below the diffraction limit, advances in HR microscopy techniques will open new perspectives for HIV-1 imaging [97,98]. These techniques require specific fluorophores. The fusion of viral proteins with small (19.4 kDa) and versatile tags such as SNAP or CLIP [99,100], that can bind these fluorophores will make it possible to optimize the labeling for each HR technique. STED microscopy provides high-speed 3D time lapse images with a lateral resolution of less than 30 nm [101,102]. Likewise, single molecule localization (SML) based techniques such as PALM [70] and STORM [64] have become more suited for many applications due to the implementation of high-speed CMOS cameras and “user-friendly” reconstruction and analysis software [103,104,105,106]. Advances in quantitative analysis of sub-diffractive objects are of particular interest for HIV-1 imaging [107]. Moreover, SML microscopy has enormous potential in single molecule tracking, as it was shown for example for Gag multimerization at the plasma membrane [108]. Tracking of individual viral proteins during viral disassembly, uncoating, or nuclear events should allow precise determination of the viral core remodeling mechanism. Likewise, additional information can be provided by using functional fluorescent probes capable of detecting environmental properties such as viscosity, polarity, membrane tension or pH [109,110,111,112,113]. Imaging of these probes conjugated to viral proteins will provide new original data on the physico-chemical characteristics of the intraviral environment and will highlight the modifications of the viral complexes linked to their remodeling during the early steps of infection.

## Figures and Tables

**Table 1:** Labeling strategies for HIV-1 components.

Viral Component	Labeling Strategy	References
Capsid	CA-TC/FlAsH, ReAsH	[27]
	Cyclophilin A-DsRed—CypA-DsRed	[17]
	CA-eGFP	[28]
	GFP-CA	[18]
Matrix	MA-GFP	[27]
Vpr	Vpr-FP	[3,26]
	mCherry-2CL-YFP-Vpr (bifunctional marker for the content release and viral core tracking; 2 protease cleavage sites are inserted between mCherry and YFP-Vpr leading to their release upon maturation)	[32]
	Vpr-QDs	[44]
Integrase	IN-TC/FlAsH	[10]
	Vpr-IN-FP (protease cleavage site inserted between Vpr and IN)	[30,31]
	Gag-IN-eGFP (protease cleavage site inserted between Gag and IN leading to release of IN-GFP upon maturation)	[29]
Nucleocapsid	NC-GFP (GFP is inserted in place of Pol in Gag-Pol, which eliminates a viral protease cleavage site downstream of NC)	[45]
	NCp7-TC/FlAsH, ReAsH	[22,36]
vRNA	APOBEC3F-FP (A3F-FP) (cytidine deaminase that is incorporated into virions during production)	[37]
	MICDDRP (multiplex immunofluorescent-cell-based detection of DNA, RNA and proteins; bDNA FISH based vDNA and vRNA labeling combined with immunostaining)	[39]
	[Ru(phen)_2_(dppz)]^2+^	[27]
	BglG and MS2 technologies (insertion of specific RNA sequences into viral genome in combination with expression of BglG or MS2 proteins fused to FP in infected cells	[12,43]
	Incorporation of 5-ethynyl uridine (EU) into vRNA during viral production followed by click labeling	[16]
vDNA	ANCHOR technology: specific DNA sequence –ANCH3 is inserted into vDNA and recognized by OR protein (derived from bacterial parB protein) fused to GFP.	[7]
	ViewHIV (bDNA-FISH combined with immunostaining of capsid)	[38]
	MICDDRP	[39]
	Incorporation of EdU into vDNA during reverse transcription followed by click labeling	[40,41]
Transcription sites	Insertion of 18 Bgl binding stem loops into the viral genome in combination with expression of BglG-mCherry in infected cells	[12,18]
Integration sites	I-Sce1 reporter system (insertion of ISce1 target site into the viral genome results in induction of I-Sce1 specific double-strand break repair activity at the site of viral integration that leads to H2AX phosphorylation. The site of integration is then detected via immunoabeling of phosphorylated H2AX histone)	[46]
Integrated DNA	CRISPR Cas9-QD: U3 region of HIV-1 proviral DNA is targeted by guide RNA and labeled by QD-conjugated Cas9 mutants lacking the endonuclease activity	[47]
Membrane/Envelope	S15-mCherry (S15 corresponds to the 15 N-terminal amino acids of p60c-SRC protein that specifically targets the cell plasma membrane. Expression of this truncated form fused to mCherry (S15-mCherry) in producer cells leads to its incorporation into the membrane of newly formed viral particles)	[48]
	DiD lipid dye (Dioctadecyl-3,3,3 3 Tetramethylindodicarbocyanine)	[49]
	EcpH-ICAM1 (GFP related pH sensor fused to intercellular adhesion molecule 1, that is incorporated into HIV-1 virions during viral assembly)	[50]
	Incorporation of modified sugars (peracylated azidomannosamine (Ac4ManNAz)) in viral envelope glycoproteins followed by click labeling	[42]
Viral Content markers	Gag-iGFP, Gag-imCherry (FP flanked by a protease cleavage site inserted between CA and MA domains of Gag)	[33,34]

**Table 2:** Summary of the main conclusions of uncoating studies with respect to the labeling strategy used. (h.p.i. hours post infection)

Capsid Labeling Strategy	Cells	Timing	Conclusions	Ref.
IF	CHO A745 HeLa	6 h.p.i. 7.5 h.p.i.	Late uncoating CA present in the nucleus	[29]
	MDMs	48 h.p.i.	No capsid loss between cytoplasm and nucleus	[75]
	SupT1-R5 (CD+T cell line)	0.5 h.p.i	Cytoplasmic loss of ~50% of CA molecules	[19]
	SupT1-R5 (CD+T cell line)	5.5 h.p.i.	Uncoating at NPC	
	MDMs	48 h.p.i.	No capsid loss between cytoplasm and nucleus	
Cyp-DsRed	HeLa derived and MDMs	<1 h.p.i.	Early cytoplasmic uncoating results in proteolytic degradation of the viral complex	[13,17,73]
	HeLa derived MDMs	~4 h.p.i. ~11.5 h.p.i.	Uncoating at the NPC with a fraction of capsid molecules entering into the nucleus with PICs	
CA-eGFP	HeLa	6 h.p.i	Uncoating at the NE CA nuclear entry independent of PICs	[28]
GFP-CA	HeLa	10.5 h.p.i.	Intact capsid enters the nucleus	[18]
